# Preparation of gastrodin‐modified dendrimer‐entrapped gold nanoparticles as a drug delivery system for cerebral ischemia–reperfusion injury

**DOI:** 10.1002/brb3.2810

**Published:** 2022-11-21

**Authors:** Wenqiang Huang, Lanlin Wang, Yanghong Zou, Xiangqian Ding, Xin Geng, Jinghui Li, Hexiang Zhao, Renli Qi, Shipeng Li

**Affiliations:** ^1^ Department of Neurosurgery First Affiliated Hospital of Kunming Medical University Kunming China

**Keywords:** anti‐inflammatory, CIRI, cytotoxicity, G5 PAMAM dendrimers, GAS, MCAO

## Abstract

**Objective:**

This study sought to evaluate the feasibility of multifunctional gastrodin (GAS)‐containing nano‐drug carrier system against cerebral ischemia–reperfusion injury (CIRI).

**Methods:**

The drug‐loaded nanocomposite (Au‐G5.NHAc‐PS/GAS) with certain encapsulation efficiency (EE) was prepared by physical adsorption method using different proportions of GAS and drug‐carrying system (Au‐G5.NHAc‐PS). High‐performance liquid chromatography was used to determine the drug loading and EE. Cultured rat astrocytes and hypothalamic neurons were assigned into four groups: PBS, Au‐G5.NHAc‐PS, Au‐G5.NHAc‐PS/GAS, and GAS. CCK‐8 assay, flow cytometry, and quantitative real‐time PCR were performed to examine the cell viability, apoptosis, and the expression of tumor necrosis factor‐α (TNF‐α), IL‐1β, and IL‐6 in the astrocytes and hypothalamic neurons, respectively. Cellular uptake of GAS and Au‐G5.NHAc‐PS/GAS was analyzed by using Hoechst 33342 staining. The animal model with focal cerebral ischemia was generated by middle cerebral artery occlusion (MCAO) in healthy male Sprague Dawley (SD) rats, and pathological changes of brain tissue and major organs in the rats were identified by hematoxylin and eosin (HE) staining. Apoptosis in rat astrocytes and hypothalamic neurons was detected by TUNEL staining and flow cytometry.

**Results:**

Au‐G5.NHAc‐PS had a spherical shape with a uniform size of 157.3 nm. Among the nanoparticles, Au‐G5.NHAc‐PS/GAS with an EE of 70.3% displayed the best release delay effect. Moreover, we observed that in vitro cytotoxicity and cellular uptake of Au‐G5.NHAc‐PS/GAS were higher than those of GAS, whereas the expression of TNF‐α, IL‐1β, and IL‐6 was significantly downregulated in Au‐G5.NHAc‐PS/GAS group as compared to G5.NHAc‐PS group. Notably, HE staining revealed that although Au‐G5.NHAc‐PS/GAS had no toxic and side effects on the main organs of rats, it alleviated the damage of brain tissue in the MCAO rats. Besides, Au‐G5.NHAc/GAS markedly reduced MCAO‐induced apoptosis.

**Conclusion:**

Au‐G5.NHAc‐PS showed favorable surface morphology, sustained drug release ability, no measurable toxicity, and good biocompatibility, indicating that GAS exerts anti‐inflammatory and antiapoptotic effects on CIRI.

## Graphical abstract

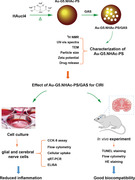

## 1 INTRODUCTION

Although stroke is a leading cause of adult disability and death worldwide, ischemic stroke is considered the main cause of death and brain tissue damage in adults (Boers et al., 2013; Feng et al., 2020). It has been shown that cerebral ischemia–reperfusion (I/R) aggravates brain damage through multiple mechanisms involving release of oxygen‐free radicals (Feng et al., 2020), overloading of Ca^2+^ and excitatory amino acids (Dong et al., 2016), and an initiation of inflammatory pathways and apoptosis (Ceulemans et al., 2010). Among these mechanisms, apoptosis and necrosis play an important role in cerebral ischemia–reperfusion injury (CIRI). Thus, it is important to understand the mechanism of brain cell apoptosis and necrosis in CIRI. Currently, thrombolytic therapy is the main option for the treatment of ischemic stroke (Li et al., 2019). However, the application of this therapy is limited due to the narrow treatment time window and potential risk of bleeding (Jin et al., 2014; Karen et al., 2014). As such, natural products extracted from Chinese herbal medicine could provide more options for the treatment of CIRI.

Gastrodin (GAS) is a natural phenolic glycoside extracted from the root of *Gastrodia elata* Blume (Liu et al., 2016; Yuan et al., 2020). GAS has been widely used in clinic to treat central nerve system disorders, such as convulsive illness, headache, dizziness, stroke, epilepsy, and amnesia (Kumar et al., 2013; Liu et al., 2018). A recent study showed that it exerts a neuroprotective effect on a rat model of subacute phase focal CIRI with middle cerebral artery occlusion (MCAO) (Liu et al., 2016). GAS has also been found to markedly reduce the content of MDA as well as the expression of tumor necrosis factor‐α (TNF‐α) and IL‐1β in the rat model of MACO I/R, indicating that it has antioxidant and anti‐inflammatory effects (Peng et al., 2015). Pharmacokinetic studies in the experimental animal models demonstrated that after entering the systemic circulation, GAS can pass through the blood–brain barrier (BBB) and then distribute rapidly in the brain (Liu et al., 2015). On the contrary, Lin et al. (2007) reported that GAS exposed to the rat brain was rather small, and the residence time of GAS in the body was very short. Therefore, the difficulty of GAS in penetrating the BBB and its short residence time in the blood circulation need to be solved prior to clinical application of GAS for the treatment of CIRI.

Poly(amide amine) (PAMAM) dendrimers have been used for a drug carrier as they are capable of extending systemic circulation time of drugs and increasing the delivery of drugs to the targets (Aisina et al., 2020). PAMAM dendrimers are a class of highly branched polymers that have been receiving extensive attention because of their monodispersity, water solubility, high drug loading, and excellent biocompatibility (Kulhari et al., 2016; Sebastien Boridy & Dusica, 2012; van Dongen et al., 2013). The dendrimer diameters and the number of surface functional groups increase with growing generation numbers (Kurtoglu et al., 2010). Although the cytotoxicity of amino‐terminated PAMAM dendrimers increases in a generation‐dependent manner, the biocompatibility of PAMAM dendrimers can be increased by appropriate hydroxyl modification (Srinageshwar et al., 2019). Generation 5 PAMAM dendrimers (G5.NH_2_) are characterized by their spherical structure, suitable size (5 nm), and appropriate size and number of terminal amino groups (Wang et al., 2017). Moreover, G5 can partially acetylate the surface charge to reduce its toxicity, and its larger interior core increases the drug‐loading percentage. As such, G5 can be used for conjugation of therapeutic or diagnostic agent (Tang et al., 2015; Yue et al., 2012).

In the present study, the surface of the G5.NH_2_ was modified by 1,3‐propane sultone (1,3‐PS) using surface amino reaction, and the gold nanoparticles were then encapsulated with sodium borohydride reduction method. Subsequently, all the remaining amino groups on the surface of the dendrimers were acetylated to improve the biocompatibility, and the drug carrier material Au‐G5.NHAc‐PS was prepared. Finally, GAS was loaded into the carrier material by the physical embedding method. Herein, we characterized the polymer structure and morphology of the nanocarriers, while determining the cytocompatibility, drug loading efficiency, and drug release‐controlling ability of Au‐G5.NHAc‐PS/GAS nanocarriers. Furthermore, we analyzed the bioactivities of drugs released from Au‐G5.NHAc‐PS/GAS nanocarriers to evaluate the potential of the nanocarriers as a drug delivery system.

## 2 METHODS

### 2.1 Materials

Male Sprague Dawley (SD) rats (aged 6–8 weeks, weighing 250–280 g) were obtained from the Hangzhou Ziyuan Experimental Animal Technology Co. Ltd. All animal procedures were approved by the Ethics Committee of Kunming Medical University and conducted in accordance with the National Institutes of Health Guide for the Care and Use of Laboratory Animals. The reagents and chemicals were purchased as follows: GAS from Kunming Pharmaceutical Corporation (Kunming, China); G5 PAMAM dendrimers (MW: 28826, surface primary amino groups: 128), 1,3‐PS, sodium borohydride (NaBH_4_), gold(III) chloride hydrate (HAuCl_4_·3H_2_O), fetal bovine serum, penicillin streptomycin, and lipopolysaccharide (LPS) from Sigma‐Aldrich Chemical Company (St. Louis, MO, USA); and RPMI‐1640 medium from GIBCO‐BRL (Life Technologies, USA). Dialyses were carried out by using Spectra/Por dialysis membranes (Spectrum Laboratories, Houston, TX).

### 2.2 Synthesis of G5.NH2‐PS

1,3‐PS and G5.NH_2_ were dissolved in water and uniformly mixed at a molar ratio of 1:20 for 3 days. To obtain G5.NH_2_‐PS, the solution was dialyzed against water with a dialysis bag (MWCO = 8000–14,000) for 3 days and then freeze‐dried.

### 2.3 Synthesis of Au‐G5.NH2‐PS

Synthesized G5.NH_2_‐PS was dissolved in ultrapure water. Under ice bath conditions, gold nanoparticles inside the G5.NH_2_ were wrapped by using HAuCl_4_ solution according to the NaBH_4_ reduction method. To obtain Au‐G5.NH_2_‐PS, the reaction mixture was mixed uniformly for 3 h, dialyzed with a dialysis bag (MWCO = 8000–14,000) for 3 days and then freeze‐fried.

### 2.4 Synthesis of Au‐G5.NHAc‐PS

Synthesized Au‐G5.NH_2_‐PS was mixed with acetic anhydride for 24 h to generate the drug‐carrying system Au‐G5.NHAc‐PS.

### 2.5 Characterization of Au‐G5.NHAc‐PS

Au‐G5.NHAc‐PS was characterized by ^1^H NMR spectroscopy and UV–vis spectrophotometry. Briefly, the Au‐G5.NHAc‐PS was dissolved in D_2_O prior to the experiments. The successful synthesis of Au‐G5.NHAc‐PS was confirmed by ^1^H NMR (Bruker Co., Billerica, MA, USA) at 300 MHz. In addition, the numbers of 1,3‐PS attached to each dendrimer were counted based on the peak area in the NMR spectrum. Then, the UV–vis spectra were detected at 520 nm by a UV–visible spectrophotometer (Thermo Scientific, USA). The particle size and zeta potential of Au‐G5.NHAc‐PS were measured with nanoparticle size analyzer and zeta potential analyzer (Zetasizer Nano ZS, Malvern), respectively. The size and morphology of gold nanoparticles in Au‐G5.NHAc‐PS were examined by transmission electron microscopy (TEM).

### 2.6 Encapsulation of GAS into Au‐G5.NHAc‐PS

Synthesized Au‐G5.NH_2_‐PS was mixed uniformly with GAS in different proportions for 24 h at room temperature to form GAS‐encapsulating Au‐G5.NHAc‐PS/GAS nanocarriers. The mixture was dialyzed with DI water using a dialysis bag (MWCO = 8000–14,000) for 1 h, and DI water was exchanged four times every hour. The free GAS in the exchanged water was determined by high‐performance liquid chromatography (HPLC). Finally, the mixture was freeze‐dried to prepare the drug delivery material (Au‐G5.NHAc‐PS/GAS) with different entrapment efficiencies (EEs).

$$\mathrm{EE}\left(\%\right)=(W_{1}-W_{2})/W_{1}\times 100$$

where *W*_1_ is the total amount of drug; *W*_2_ is the amount of free drug.

### 2.7 In vitro drug release kinetics

GAS release kinetics from Au‐G5.NHAc‐PS/GAS nanocarriers was analyzed in PBS (10 mM, pH7.4). The solution of the nanocarriers was transferred to dialysis bags (MWCO = 8000–14,000 Da), and the sample bags were then immersed into 14 ml of fresh release media PBS (pH 7.4). At predetermined time points, the release medium was collected, and an equal volume of fresh PBS was added. The amount of GAS released at various time points was quantified by HPLC analysis. All experiments were performed in triplicate.

### 2.8 Cell culture

Rat astrocytes (Cat No.: CP‐R137) and hypothalamic neurons (Cat No.: CP‐R239) were obtained from Procell Life Science & Technology Co., Ltd. (Wuhan, Hubei, China). The following four groups were included in the experiments: blank control (treated with PBS only) and three experimental groups (treated with GAS, Au‐G5.NHAc‐PS, and Au‐G5.NHAc‐PS/GAS, respectively). Cells were cultured in RPMI 1640 medium supplemented with 10% fetal bovine serum, 100 mg/ml streptomycin, and 100 U/ml penicillin at 37°C in a humidified incubator with 5% CO_2_.

### 2.9 CCK‐8 assay

The cell viability was evaluated by CCK‐8 assay. Rat astrocytes or hypothalamic neurons (1 × 10^5^ cells per well) were seeded into 96‐well plates and allowed to adhere for 24 h at 37°C. Afterward, the culture medium was replaced by fresh medium containing GAS, Au‐G5.NHAc‐PS, or Au‐G5.NHAc‐PS/GAS, and the cells were grown for another 48 h at 37°C. The medium was then aspirated, and cells were washed thrice with PBS. For the assay, 10 μl of CCK‐8 reagent was added to each well, and the 96‐well plate was subsequently incubated for 1 h at 37°C in the dark. The absorbance value was measured at 450 nm using a microplate reader (Thermo Fisher Scientific, USA). Each assay was repeated six times.

### 2.10 Flow cytometry

Apoptosis in the cells treated with GAS, Au‐G5.NHAc‐PS, or Au‐G5.NHAc‐PS/GAS was examined by Annexin V‐FITC/PI double labeling to determine their cytotoxicity. Briefly, cells were digested with 0.25% trypsin and suspended in 500 μl binding buffer at a concentration of 1 × 10^6^ cells/ml. Afterward, the cells were resuspended in 500 μl binding buffer and then stained with 5 μl V‐FITC Annexin and 5 μl PI solutions for 15 min in the dark at room temperature. Apoptosis was analyzed by using a flow cytometer (BD Biosciences, CA, USA). Each assay was performed at least three times.

### 2.11 Cellular uptake

A fat‐soluble red fluorescent DID dye was used as a model drug to replace GAS for the sake of lack of fluorescence. To investigate the cellular uptake of GAS in glial cells, DID‐loaded Au‐G5.NHAc‐PS nanoparticles were prepared by the methods described above. Glial cells (1 × 10^5^ cells/well) were seeded in 24‐well plates, grown overnight, and then incubated with DID (10 ng/ml) and Au‐G5.NHAc‐PS/DID nanoparticles (Dou et al., 2019; Strachan et al., 2020). After 24 h of incubation, the cells were collected and rinsed three times with PBS to remove DID on the cell surface. Thereafter, cells were fixed with 4% paraformaldehyde for 30 min at room temperature. After fixation, cells were gently rinsed three times with PBS and stained with Hoechst 33342 for 30 min at room temperature. Finally, cells were rinsed with PBS and mounted with 90% glycerol. Cell morphology was observed under a fluorescence microscope (Olympus Corporation, Japan).

### 2.12 Anti‐protein absorption

A certain amount of Au‐G5.NHAc‐PS or Au‐G5.NHAc‐PS/GAS and 10 ml of BSA‐containing buffer were added to an Erlenmeyer flask. The flask was then placed in a shaker (100 r/min) at 25°C. After 24 h of adsorption, the supernatant was taken and UV absorption spectra of Au‐G5.NH2‐PS and Au‐G5.NH2‐PS/GAS were recorded at 278 nm by an ultraviolet spectrophotometer.

### 2.13 Anti‐inflammatory assay

Glial or nerve cells were seeded in a 24‐well plate at a density of 1 × 10^6^ cells per well and incubated with LPS at a concentration of 100 ng/ml for 3 h. After the incubation, cells were treated with 1 ml of medium‐containing Au‐G5.NHAc‐PS or Au‐G5.NHAc‐PS/GAS for 12 h. Thereafter, cells were washed gently with the warm medium and replenished with fresh medium containing 100 ng/ml of LPS for an incubation of 24 h. The control group was treated with Au‐G5.NHAc‐PS or Au‐G5.NHAc‐PS/GAS, and the one without LPS induction and treatment was used as the negative control group. At the same time, the treatment with 100 ng/ml LPS was also studied as the positive control. After 24 h, the culture medium was sampled and centrifuged at 1500 rpm for 5 min. The supernatant was collected and stored at −80°C for further assays.

### 2.14 Quantitative real‐time PCR (qRT‐PCR)

Rat astrocytes and hypothalamic neurons were randomly divided into six groups: control group, Au‐G5.NHAc‐PS group, Au‐G5.NHAc‐PS/GAS group, LPS group, LPS + Au‐G5.NHAc‐PS group, and LPS + Au‐G5.NHAc‐PS/GAS group. Total RNA was isolated from the cells with Trizol reagent (Thermo Fisher Scientific, Waltham, MA, USA), quantified by using UV–visible spectrophotometer and reverse‐transcribed into cDNA. Quantitative real‐time PCR (qRT‐PCR) was then performed by using SYBR Premix Ex Taq (Takara, Japan) according to the manufacturer's instructions. Thermal cycler conditions were as follows: 95°C for 3 min followed by 40 cycles of 95°C for 7 s and 57°C for 10 s. Afterward, PCR products were heated to 72°C for 15 s, and melting curve analysis was performed to confirm their authenticity. The differences in critical thresholds between target genes and 18S rRNA were used to normalize the results in each assay. The sequences of primers used in the study are listed in Table 1.

**TABLE 1:** The primer sequences for quantitative real‐time PCR (qRT‐PCR)

Gene	Primers
TNF‐α	F:5′‐AACACACGAGACGCTGAAGT‐3′
	R:5′‐TCCAGTGAGTTCCGAAAGCC‐3′
IL‐1β	F:5′‐GGCTTCCTTGTGCAAGTGTC‐3′
	R:5′‐CACACACTAGCAGGTCGTCA‐3′
IL‐6	F:5′‐ACCCCAACTTCCAATGCTCT‐3′
	R:5′‐GGATGGTCTTGGTCCTTAGCC‐3′
β‐actin	F:5′‐GCAGGAGTACGATGAGTCCG‐3′
	R:5′‐ACGCAGCTCAGTAACAGTCC‐3′

Abbreviation: TNF‐α, tumor necrosis factor‐α.

### 2.15 Establishment of rat model with middle cerebral artery occlusion (MCAO) and animal grouping

For the model establishment, 25 rats were first anesthetized with isoflurane (3% for induction and 1.5% for maintenance) in oxygen‐enriched air using a facemask, and the body temperature was maintained at 37°C by a heating pad. The rats were fixed, and an incision was made along the midline of the neck to expose the right common carotid artery (CCA), external carrot artery, and internal carotid artery (ICA). The proximal ends of the CCA were clamped with a micro‐arterial clamp, and a small incision was made at the bifurcation of CCA and ICA. Thereafter, the thread plug was inserted into the beginning of the middle cerebral artery (MCAO) through the incision to embolize MCA. Although a small incision was made in CCA, a wire plug (0.25 mm; RWD Life Science Co., Ltd., Shenzhen, China) was gently inserted into ICA. Finally, the distal end of CCA was ligated, and the wound was sutured. MCA was occluded for 2 h and then subjected to reperfusion for 5 days. The ischemic insult was evaluated based on neurological deficits characterized by serve contralateral hemiparesis. MCAO modeling process is shown in Figure S1A.

Three rats died during the modeling process. A total of 15 male SD rats were randomly assigned into 5 groups with 3 for each: the sham group, MCAO group, MCAO + Au‐G5.NHAc‐PS group, MCAO + GAS group, and MCAO + Au‐G5.NHAc‐PS/GAS group. The body weight was almost the same for rats in each group, and all animals were in a good health (Figure S1B). In the sham group, skin and related tissues of rats were cut to expose the right carotid artery system, but no suture was inserted. Rats in the MCAO group were intraperitoneally injected with NaCl solution (0.9% w/v, 0.2 ml for each). Meanwhile, rats in the remaining three groups were intravenously administered through the tail vein with Au‐G5.NHAc‐PS (40 mg/kg), GAS (100 mg/kg), or Au‐G5.NHAc‐ps/GAS (40 mg/kg) three times within 7 days. All animals were housed at constant room temperature (23 ± 2°C, 60%–65% humidity) under a 12‐h dark/light cycle, with free access to food and water.

### 2.16 In vivo toxicity

At the end of the experiment, rats were deeply anesthetized with ether, and the perfusion–fixation was performed. The brains were removed, snap‐frozen in liquid nitrogen and then fixed in 4% paraformaldehyde overnight at 4°C. In the meantime, organs, including liver, lung, heart, kidney, and spleen, were harvested and fixed in a 10% formalin solution (Pishavar et al., 2019). Afterward, fixed organs were dehydrated and embedded with paraffin. The paraffin‐embedded samples were then cut into 4‐μm sections using a conventional microtome. The sections were stained with hematoxylin and eosin (HE), and all images were captured at 100× magnification using an optical microscope. The TUNEL staining was performed to detect apoptosis in brain tissue, and the images at 200× magnification were captured. In addition, the brain tissue was dissociated, and the cell suspension was prepared for apoptosis detection by flow cytometry.

### 2.17 Statistical analysis

Experimental results were presented as the mean ± standard deviation (SD). At least three experimental replicates were performed for each sample. The significance of the experimental data was evaluated by PRISM GraphPad 8.0 software, *t*‐tests, one‐way or two‐way analysis of variance followed by Tukey's post hoc tests. The level of statistical significance was set at *p* < .05, and *p*‐values were indicated accordingly.

## 3 RESULTS

### 3.1 Characterization of Au‐G5.NHAc‐PS

**FIGURE 1:**
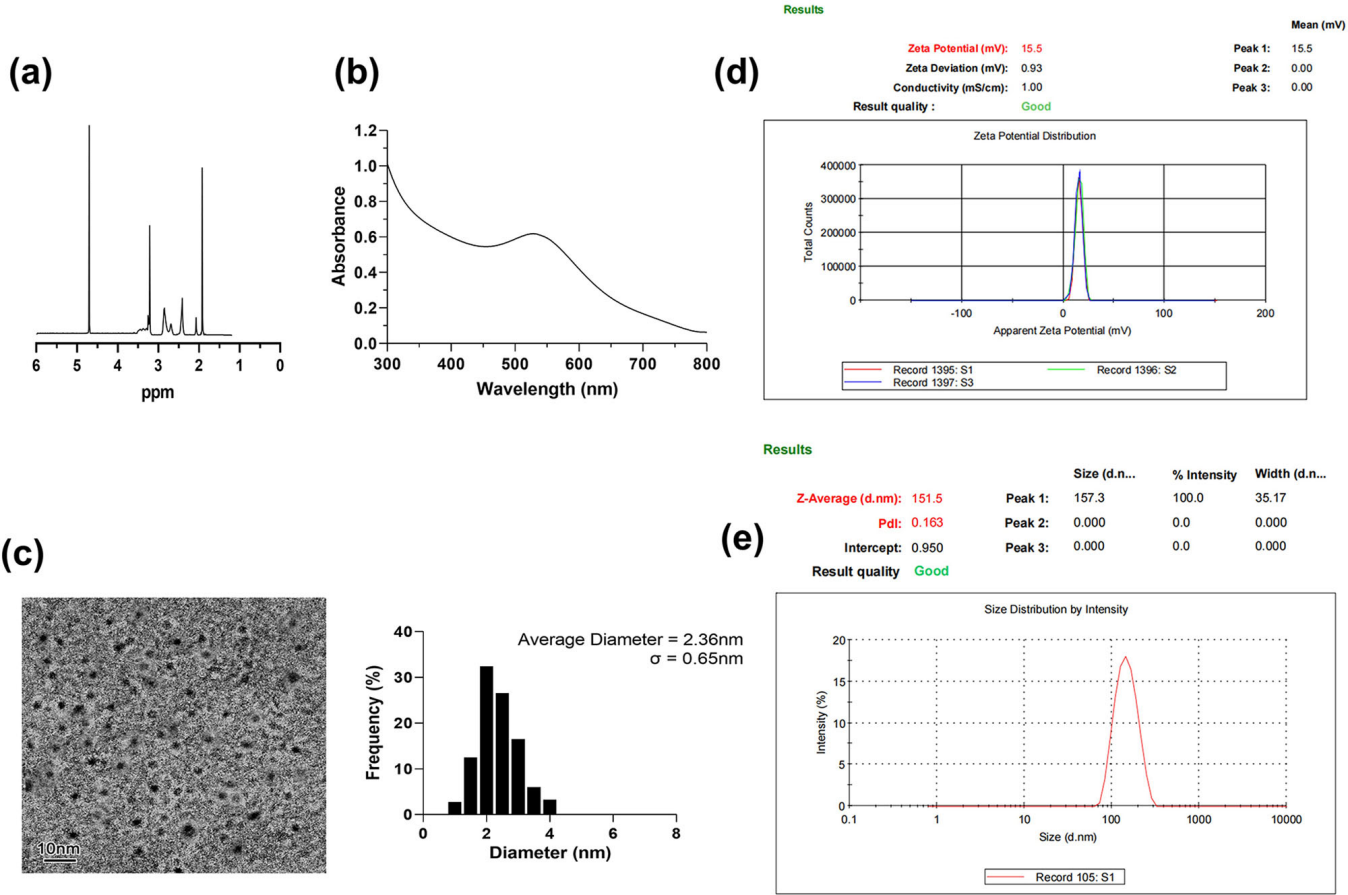
(a) ^1^H‐NMR spectra of Au‐G5.NHAc‐PS; (b) characterization of gold nanoparticles in Au‐G5.NHAc‐PS with UV–vis adsorption spectra; (c) transmission electron microscopy (TEM) image and size distribution of gold nanoparticles in Au‐G5.NHAc‐PS; (d and e) the zeta potential and particle size of Au‐G5.NHAc‐PS

As depicted in Figure 1a, Au‐G5.NHAc‐PS was synthesized and characterized by ^1^H NMR spectrum. The methylene proton peak of G5‐PAMAM was between 2.2 and 3.4 ppm, indicating that G5‐PAMAM was successfully synthesized and characterized. The number of grafting of 1,3‐PS monomers onto G5 PAMAM was calculated by integrating the areas under peaks at 2.2 and 3.4 ppm and was found to be approximately 17.6. In addition, the signal at 1.9 ppm represented the acetyl group that was added onto G5‐PAMAM.

We further characterized the Au‐G5.NHAc‐PS by UV–vis spectrometry and TEM. As shown in Figure 1b, Au‐G5.NHAc‐PS displayed a typical surface plasmon resonance peak at 520 nm, indicative of the successful synthesis of gold nanoparticles. Moreover, TEM observation revealed that the gold nanoparticles had a spherical shape with a mean diameter of 2.36 nm, showing a good dispersion of Au‐G5.NHAc‐PS (Figure 1c). All these results demonstrated that the modification on the surface of G5 PAMAM dendrimers caused no alterations in the morphology and structure of the nanoparticles. To analyze the surface charge of Au‐G5.NHAc‐PS nanoparticles, we measured the zeta potential. As illustrated in Figure 1d, the nanoparticles had a positive potential of 15.5 ± 0.93 mV, suggesting the presence of amine groups in Au‐G5.NHAc‐PS. In the meantime, the particle size of Au‐G5.NHAc‐PS was found to be 151.5 nm (Figure 1e).

### 3.2 In vitro release

**FIGURE 2:**
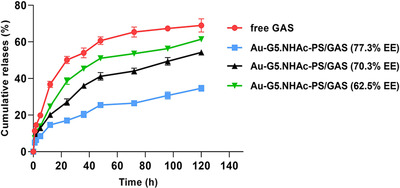
In vitro release profiles of gastrodin (GAS) from Au‐G5.NHAc‐PS/GAS conjugates in PBS (pH7.4) at 37°C under different EEs (*n* = 3)

The EE of three different groups of Au‐G5.NHAc‐PS/GAS nanoparticles prepared by physical dialysis were 77.3%, 70.3%, and 62.5%, respectively. To investigate the in vitro drug release profiles of GAS and Au‐G5.NHAc‐PS/GAS, we measured the free GAS that was released outside the dialysis bag at specific time intervals under a simulated physiological condition (pH 7.4). As shown in Figure 2, the initial burst release of free GAS from the nanoparticles occurred in the first hour, reaching 11.37% ± 0.73%. The content of GAS released from the nanoparticles with an EE of 77.3%, 70.3%, and 62.5% at the first hour was found to be 4.95% ± 0.21%, 6.67% ± 0.27%, and 7.34% ± 0.27%, respectively. Among the three groups of nanoparticles, Au‐G5.NHAc‐PS/GAS with an EE of 77.3% had a lowest cumulative release of GAS within the first hour, whereas there was no significant difference in the release between nanoparticles with an EE of 70.3% and those with an EE of 62.5%. Notably, the release of GAS decreased significantly within 24 h as the EE increased (*p* < .05). She et al. (2013) demonstrated that the drug nanocarriers need high stability, which ensures a long‐term gradual release of drugs in the blood circulation. The present study showed that Au‐G5.NHAc‐PS/GAS nanoparticles had a potential in controlling the release of GAS over a long period of 120 h. Moreover, 24 h later, GAS was continuously released from Au‐G5.NHAc‐PS/GAS and accumulated in cells. These observations suggested that Au‐G5.NHAc‐PS/GAS delivery system could reduce the release of free GAS in the blood circulation before reaching the damage sites. Taken together, we proposed that the nano drug delivery system Au‐G5.NHAc‐PS/GAS had the best release delay effect at an EE of 70.3%.

### 3.3 In vitro cytotoxicity

**FIGURE 3:**
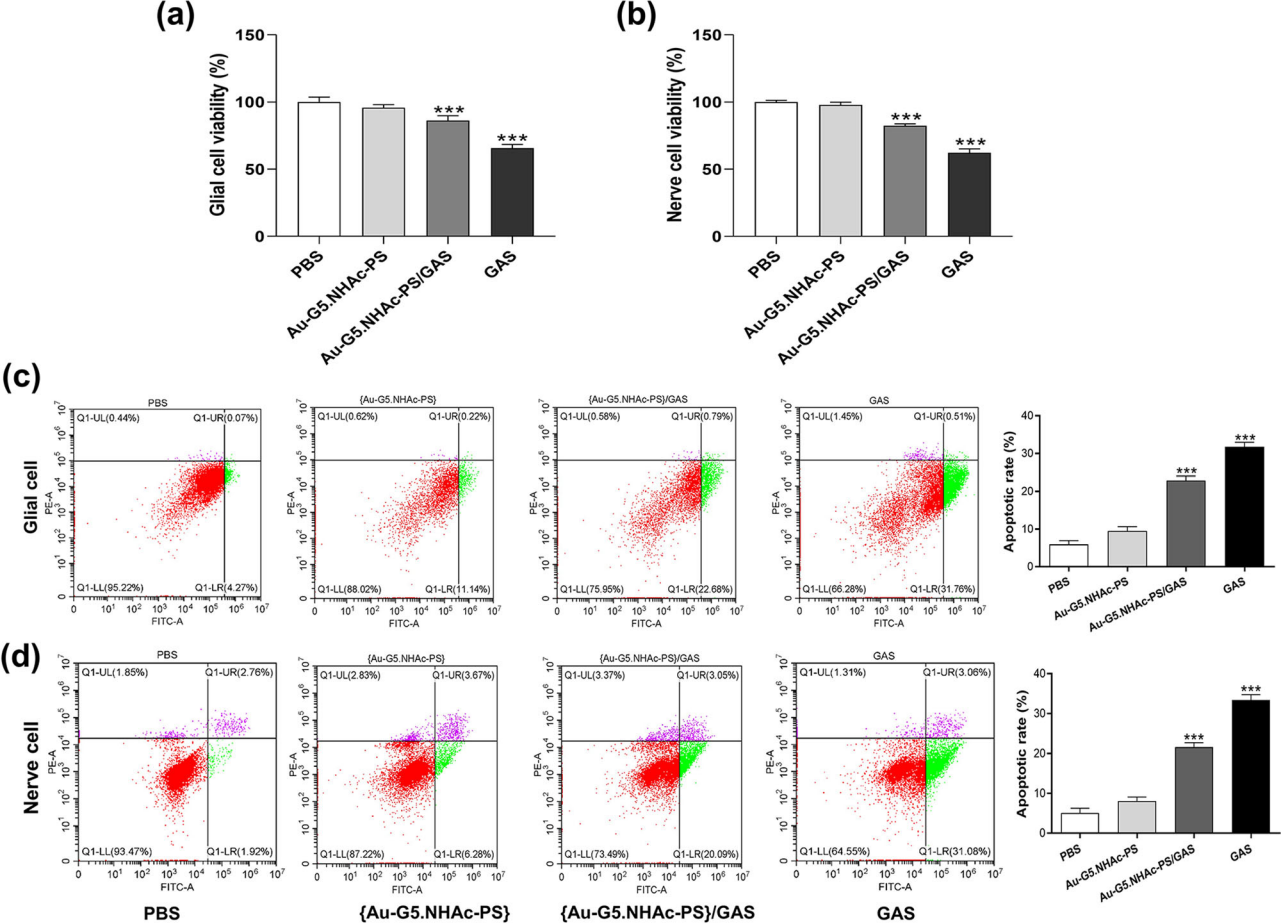
(a and b) CCK‐8 assay‐based analysis of cell viability in Rat astrocytes and hypothalamic neurons treated with Au‐G5.NHAc‐PS or Au‐G5.NHAc‐PS/GAS; (c and d) flow cytometric analysis of the apoptosis rate in glial cells and nerve cells. ***p < .0001 compared with the PBS group

The in vitro cytotoxicity of Au‐G5.NHAc‐PS and Au‐G5.NHAc‐PS/GAS against glial and nerve cells was determined by CCK‐8 assay. As shown in Figure 3a,b, there was no significant difference in the cytotoxicity between the PBS group and Au‐G5.NHAc‐PS/GAS group (*p* > .05, *n* = 6). Compared with Au‐G5.NHAc‐PS group, a significant decrease in the cell viability was observed in both GAS and Au‐G5.NHAc‐PS/GAS groups (*p* < .0001, *n* = 6). These data indicated that although Au‐G5.NHAc‐PS had an excellent biocompatibility, free GAS showed significant toxicity toward both glial cells and cerebral nerve cells.

We next performed a quantitative analysis of apoptosis by flow cytometry. As depicted in Figure 3c,d, the apoptosis index of rat astrocytes and hypothalamic neurons treated with GAS or Au‐G5.NHAc‐PS/GAS was significantly higher than that of those cells treated with PBS or Au‐G5.NHAc‐PS (*p* < .0001). Meanwhile, decreased apoptosis was detected in Au‐G5.NHAc‐PS/GAS group as compared to free GAS group. Consistent with the CCK‐8 assay, flow cytometry analysis indicated that Au‐G5.NHAc‐PS could significantly reduce the in vitro cytotoxicity of GAS.

### 3.4 Phagocytosis assay

#### 3.4.1 Cellular uptake

**FIGURE 4:**
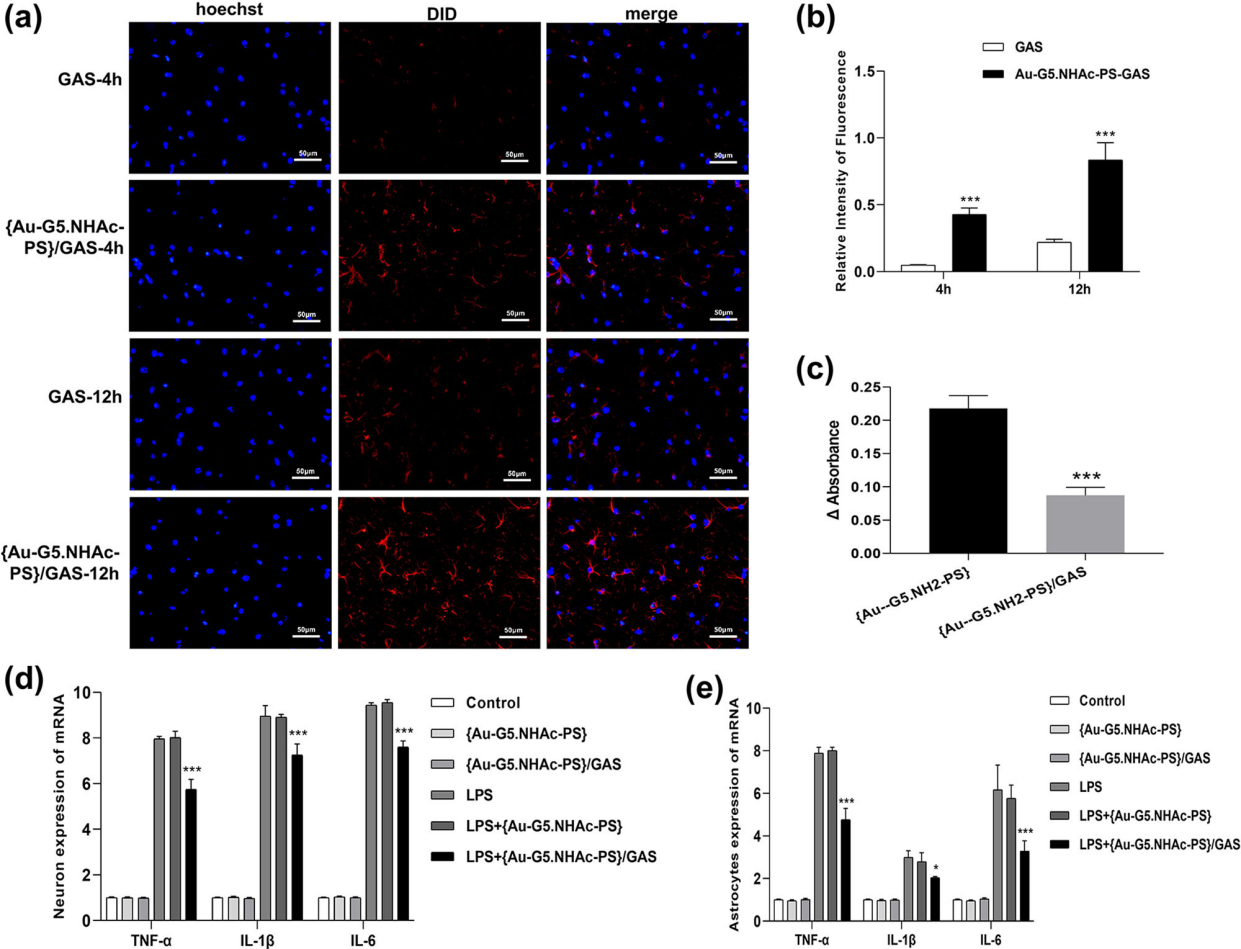
Uptake of gastrodin (GAS) and Au‐G5.NHAc‐PS/GAS by glial cells at different time points following the cell transfection: (a) fluorescent confocal microscopy images showing the cellular uptake. Scale bar = 50 μm; (b) the relative fluorescence intensity showing the extent of cellular uptake. ***p < .0001 compared with GAS group; (c) anti‐protein adsorption of Au‐G5.NHAc‐PS and Au‐G5.NHAc‐PS/GAS. ***p < .0001 compared with Au‐G5.NHAc‐PS group

To investigate the relationship between cells and Au‐G5.NHAc‐PS/GAS, we prepared DID‐loaded Au‐G5.NHAc‐PS nanoparticles for the assays. As presented in Figure 4a,b, although a cellular uptake of both GAS and Au‐G5.NHAc‐PS/GAS was observed in glial cells, a significant increase in the uptake was found in Au‐G5.NHAc‐PS/GAS group compared with GAS group (*p* < .0001). Moreover, cellular uptake of both GAS and GAS combined with Au‐G5.NHAc‐PS nanoparticles displayed a time‐dependent manner. All these results suggested that Au‐G5.NHAc‐PS could facilitate cellular uptake of GAS. This effect could be attributed to the interactions between negatively charged cell membranes and positively charged nanoparticles.

#### 3.4.2 Anti‐protein adsorption

Unlike BSA protein, G5 has no characteristic UV absorption peaks at 278 nm. We, therefore, sought to determine the anti‐protein adsorption effects of Au‐G5.NHAc‐PS and Au‐G5.NHAc‐PS/GAS. To this end, we calculated differences in UV absorption at 278 nm between Au‐G5.NHAc‐PS and Au‐G5.NHAc‐PS/GAS before and after the incubation with BSA protein. As shown in Figure 4c, UV absorption value of Au‐G5NHAc‐PS/GAS was significantly lower than that of Au‐G5.NHAc‐PS, suggesting that Au‐G5.NHAc‐PS may prolong the persistence of GAS in the blood circulation.

#### 3.4.3 The cytokine release related to Au‐G5.NHAc‐PS and Au‐G5.NHAc‐PS/GAS

**FIGURE 5:**
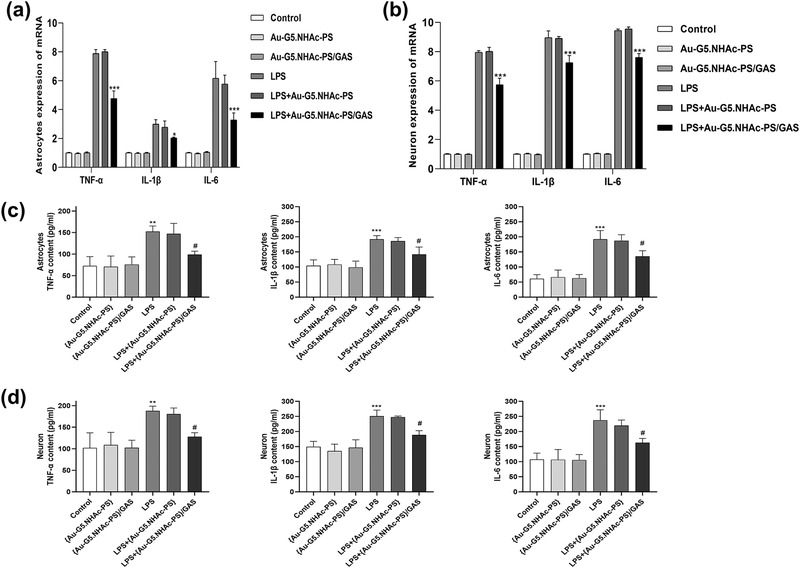
Effects of Au‐G5.NHAc‐PS and Au‐G5.NHAc‐PS/GAS on (a and b) mRNA and (c and d) protein expression levels of tumor necrosis factor‐α (TNF‐α), IL‐1β, and IL‐6 in glial and cerebral nerve cells before and after lipopolysaccharide (LPS) induction. *p < .05, ***p < .0001 compared with Au‐G5.NHAc‐PS group

Upon activation, rat astrocytes and hypothalamic neurons in the healthy brain release a large number of inflammatory factors (Yao et al., 2019). To investigate the anti‐inflammatory activity of Au‐G5.NHAc‐PS/GAS, glial and cerebral nerve cells were pretreated with LPS (10 μg/ml), followed by treatment with Au‐G5.NHAc‐PS or Au‐G5.NHAc‐PS/GAS. As illustrated in Figure 5a–d, there were no significant differences in mRNA and protein expression of TNF‐α, IL‐1β, and IL‐6 between the control group and Au‐G5.NHAc‐PS or Au‐G5.NHAc‐PS/GAS group (*p* > .05). Notably, LPS treatment resulted in a significant increase in the expression of the inflammatory factors at both mRNA and protein levels. Moreover, we found that although there were no statistical differences in the levels of TNF‐α, IL‐1β and IL‐6 between LPS and LPS + Au‐G5.NHAc‐PS groups (*p* > .05), a markedly reduced release of cytokines was detected in LPS + Au‐G5.NHAc‐PS/GAS group as compared to LPS group (*p* < .05). Clearly, it needs to be determined whether these observations are related to a decline in the cell viability.

### 3.5 In vivo antitumor activity

**FIGURE 6:**
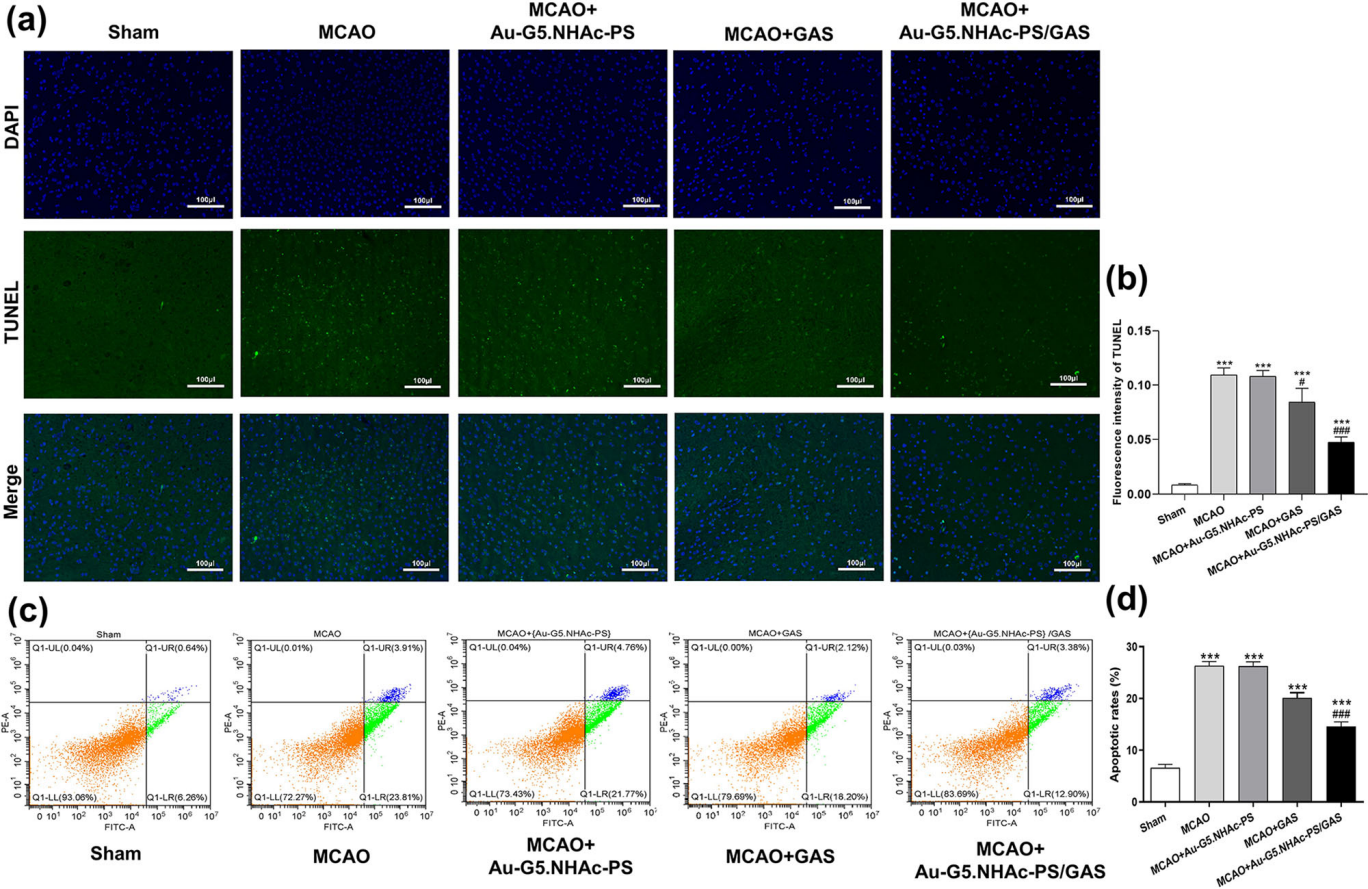
(a) Histological analysis of antiapoptotic effects of GAS, Au‐G5.NHAc‐PS, and Au‐G5.NHAc‐PS/GAS in middle cerebral artery occlusion (MCAO) rats. Scale bar = 100 μm; (b) fluorescence quantitative analyses of TUNEL staining; (c) apoptosis in rat astrocytes and hypothalamic neurons was measured by flow cytometry assay; (d) quantitative data of the apoptotic rates in the astrocytes and hypothalamic neurons. ***p < .0001 compared with the sham group, #*p* < .05 and ###*p* < .0001 compared with the MCAO group

We performed TUNEL assay to determine anti‐apoptotic effects of GAS. As shown in Figure 6a, no apoptosis was detected in brain tissue of the sham group, whereas there were a large number of apoptotic cells in both MCAO and MCAO + Au‐G5.NHAc‐PS groups. Strikingly, fewer apoptotic cells were observed in MCAO + GAS or MCAO + Au‐G5.NHAc‐PS/GAS group as compared to the MCAO group. Moreover, the TNUEL assay showed that although a significant decrease in the apoptosis level was found in sections treated with Au‐G5.NHAc‐PS/GAS compared with the sham group (*p* < .0001), MCAO + GAS group displayed less apoptosis than the MCAO group (*p* < .0001) (Figure 6b).

We further examined the apoptosis by Annexin V‐FITC/PI double staining assay. As depicted in Figure 6c,d, the rate of apoptosis in brain tissue was significantly increased in MCAO, MCAO + GAS, MCAO + Au‐G5.NHAc‐PS, and MCAO + Au‐G5.NHAc‐PS/GAS groups compared with the sham group (*p* < .0001). Moreover, although there was no significant difference in the apoptotic rate between MCAO and MCAO + Au‐G5.NHAc‐PS groups (*p* > .05), the apoptotic rate in MCAO + Au‐G5.NHAc‐PS/GAS group was markedly lower than that in both the sham and MCAO groups (*p* < .0001). These data further suggested that Au‐G5.NHAc‐PS may prolong the persistence of GAS in the blood circulation.

### 3.6 Histological analysis of brain and other major organs

**FIGURE 7:**
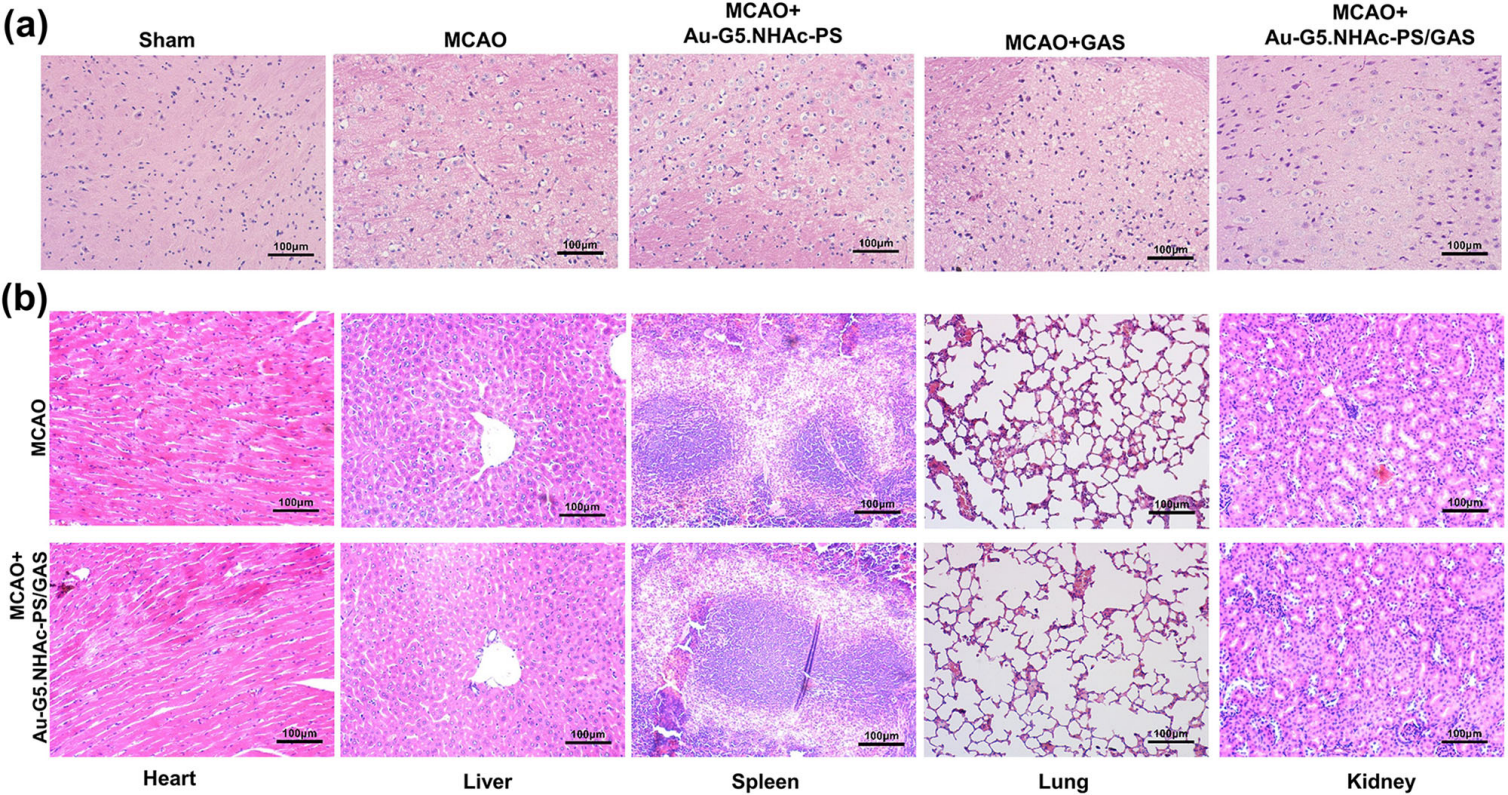
(a) Hematoxylin and eosin (HE) staining‐based histological examination of mouse brain tissue. Scale bar = 100 μm; (b) HE staining of tissue sections of the major mouse organs. Scale bar = 100 μm

To examine the potential toxicity of GAS, Au‐G5.NHAc‐PS and Au‐G5.NHAc‐PS/GAS in rats, we performed HE staining on major organs, including brain, heart, liver, spleen, lung, and kidney. As shown in Figure 7a, cerebral nerve cells appeared normal in the sham group, whereas severe cellular edema, cell degeneration, and necrosis were observed in the white matter of cerebral cortex in the MCAO group. Moreover, the MCAO group exhibited a disordered arrangement of cortical granule cells, abnormal glial cell proliferation, and the presence of disorganized nerve fibers in the form of nets and cords. Notably, all these alterations were relieved in MCAO + GAS or MCAO + Au‐G5.NHAc‐PS/GAS groups, whereas a more significant improvement was present in MCAO + Au‐G5.NHAc‐PS/GAS group in comparison with MCAO + GAS group. In the meantime, observation with optical microscope revealed no significant alterations in the morphology of other major organs, including cell arrangement, intercellular space, and fibrosis in MCAO + Au‐G5.NHAc‐PS/GAS group compared with MCAP group (Figure 7b). Together, these results indicated that Au‐G5.NHAc‐PS/GAS had a good biocompatibility.

## 4 DISCUSSION

Although ischemic stroke seriously threatens human life and health, cerebral I/R could further aggravate brain damage (Liu et al., 2016). Although there are many drugs for the treatment of CIRI, the presence of the BBB makes the treatment more difficult. In this study, we modified the surface group of G5 PAMAM to generate a nano drug‐carrying system Au‐G5.NHAc‐PS with proper encapsulation capacity and BBB penetrating ability, while preparing the drug‐carrying system Au‐G5.NHAc‐PS/GAS loaded with GAS by physical adsorption method. Previous studies have demonstrated that nanoparticles can enhance drug penetration through the BBB into the brain (Jain et al., 2016; Zhao et al., 2015). We further showed that Au‐G5.NHAc‐PS/GAS significantly reduced cell proliferation and increased the apoptosis level in the models of rat astrocytes and hypothalamic neurons. Besides, Au‐G5.NHAc‐PS/GAS markedly downregulated the expression of immune factors in LPS‐induced astrocytes and hypothalamic neurons.

Cerebral nerve damage or death usually leads to an excessive activation of glial cells (Ying et al., 2015), which may increase the expression of iNOS and release of NO (Zou et al., 2014), thus inducing TNF‐α as well as highly detrimental neurotoxicity (Yao et al., 2019). In the current study, CCK‐8 assay and flow cytometry analysis demonstrated that a certain concentration of Au‐G5.NHAc‐PS was noncytotoxic. However, previous studies have shown that GAS had the property of low toxicity in clinic (Yuan et al., 2018; Zhang et al., 2018), so free‐GAS and Au‐G5.NHAc‐PS/GAS have more significant cytotoxicity than Au‐G5.NHAc‐PS. Hoechst 33342 staining revealed that the GAS and Au‐G5.NHAc‐PS/GAS were uptaken by rat astrocytes and hypothalamic neurons. Au‐G5.NHAc‐PS/GAS exhibited a significantly higher cellular uptake than GAS, indicating that Au‐G4.NHAc‐PS can promote the cellular uptake of GAS. It has also been shown that GAS possesses antioxidant, anti‐inflammatory, and anti‐apoptotic effects, which contribute to protecting brain from CIRI (Liu et al., 2018). Notably, an activation of rat astrocytes and hypothalamic neurons can lead to the production of pro‐inflammatory cytokines, causing stroke damage (Jin et al., 2015). Herein, we examined the expression of inflammatory factors in the cells to evaluate the immunogenicity of Au‐G5NHAc‐PS and Au‐G5.NHAc‐PS/GAS. The qRT‐PCR assay showed that both Au‐G5.NHAc‐PS and Au‐G5.NHAc‐PS/GAS displayed no immunogenicity, suggesting that these nanoparticles could be used as good drug‐loading carriers. In the meantime, we observed that Au‐G5.NHAc‐PS/GAS suppressed the expression of TNF‐α, IL‐1β, and IL‐6 in LPS‐activated glial and cerebral nerve cells by inhibiting β‐actin signaling pathway. Consistent with the previous studies (Peng et al., 2013; Yao et al., 2019), the findings in our study provided evidence that GAS effectively inhibits the expression of TNF‐α, IL‐1β, and IL‐6 in rat astrocytes and hypothalamic neurons. Given that GAS has been found to reduce cerebral I/R in rats by inhibiting the expression of TNF‐α, IL‐1β, and IL‐6, we speculate that GAS may inhibit the expression of anti‐inflammatory cytokines by increasing cytotoxicity.

Although inflammatory pathways and apoptosis are initiated in the subacute stage of cerebral ischemia, this initiation usually leads to the death of nerve cells (Ceulemans et al., 2010). It has been reported that the expression of iNOS during cerebral ischemia could further destroy the BBB and cause brain edema (Li et al., 2015). In this study, we established the MCAO model to comparatively analyze the damage of ischemic nerve cells before and after the treatment. Meanwhile, HE staining, TUNEL assay and flow cytometry analysis were performed to determine side effects of GAS‐containing Au‐G5.NHAc‐PS on the major organs of rats and apoptosis in the astrocytes and hypothalamic neurons, respectively. Here, we found that Au‐G5.NHAc‐PS/GAS relieved brain tissue damage and inhibited cell apoptosis, suggesting a certain therapeutic effect of Au‐G5.NHAc‐PS/GAS on CIRI. Wang et al. (2021) showed that the encapsulation of the antioxidant asparagine in nanoprobe‐loaded microbubbles can facilitate drug delivery to the brain, promoting BBB opening and inhibiting apoptosis. In the present study, histological analysis revealed edema, degeneration, necrosis, and disordered arrangement of cells and nerve fibers in the sebaceous layer of MCAO mouse brain. Notably, we observed that although treatment with GAS or Au‐G5.NHAc‐PS/GAS alleviated those histological abnormalities, the abnormalities in the GAS group were slighter than those in Au‐G5.NHAc‐PS/GAS group. These observations may be related to effects such as environmental factors, which could be unavoidable during the experiment. Consistently, it has been demonstrated that asparagine significantly improved brain edema and neurological deficits after cerebral hemorrhage (Liu et al., 2020). In the current study, no significant alterations in the morphology of major organs were detected in the Au‐G5.NHAc‐PS/GAS group (Pishavar et al., 2019). Taken together, these results indicate that the drug delivery system Au‐G5.NHAc‐PS/GAS prepared by the modification of G5 PAMAM dendrimers has a good biocompatibility and barely affects biological function of the major organs. Besides, TUNEL staining and flow cytometry analysis showed that although both GAS and Au‐G5.NHAc‐PS/GAS had anti‐apoptotic effects on CIRI, the effects of Au‐G5.NHAc‐PS/GAS were more significant. These observations demonstrated that compared with GAS, the drug‐loading system Au‐G5.NHAc‐PS/GAS had an increased BBB penetrating ability.

## 5 CONCLUSION

In this study, we prepared a drug‐carrying system Au‐G5.NHAc‐PS by modifying G5 PAMAM. We showed that Au‐G5.NHAc‐PS had a relatively good performance, as indicated by high transfection rate, high cellular uptake, and low cytotoxicity. Meanwhile, in vivo and in vitro studies revealed that compared with unmodified G5 PAMAM, the modified G5 PAMAM dendrimers markedly improved the therapeutic effects of GAS on CIRI. All these data suggest that the Au‐G5.NHAc‐PS may potentially serve as a drug delivery system for GAS treatment of CIRI.

## AUTHOR CONTRIBUTIONS

Wenqiang Huang and Shipeng Li conceived and designed the experiments. Lanlin Wang, Yanghong Zou, and Xiangqian Ding performed the experiments. Xin Geng, Jinghui Li, and Hexiang Zhao analyzed the data. Wenqiang Huang interpreted the results and wrote the manuscript. Shipeng Li contributed to the review of the manuscript. All authors read and approved the final manuscript.

## CONFLICT OF INTEREST

The authors declare that they have no conflict of interest.

## CONSENT FOR PUBLICATION

All authors have read and approved the final manuscript.

### PEER REVIEW

The peer review history for this article is available at https://publons.com/publon/10.1002/brb3.2810

## Supporting information

FIGURE S1 (A) the flowchart of MCAO modeling in rats; (B) trends in body weight of rats in each group

## Data Availability

The analyzed datasets generated during this study are available from the corresponding author on reasonable request.
