# Supplementary figures and images for: Preparation of gastrodin‐modified dendrimer‐entrapped gold nanoparticles as a drug delivery system for cerebral ischemia–reperfusion injury
Source: Brain Behav. 2022 Nov 21;12(12):e2810. doi: 10.1002/brb3.2810 (PMC9759136; doi:10.1002/brb3.2810)

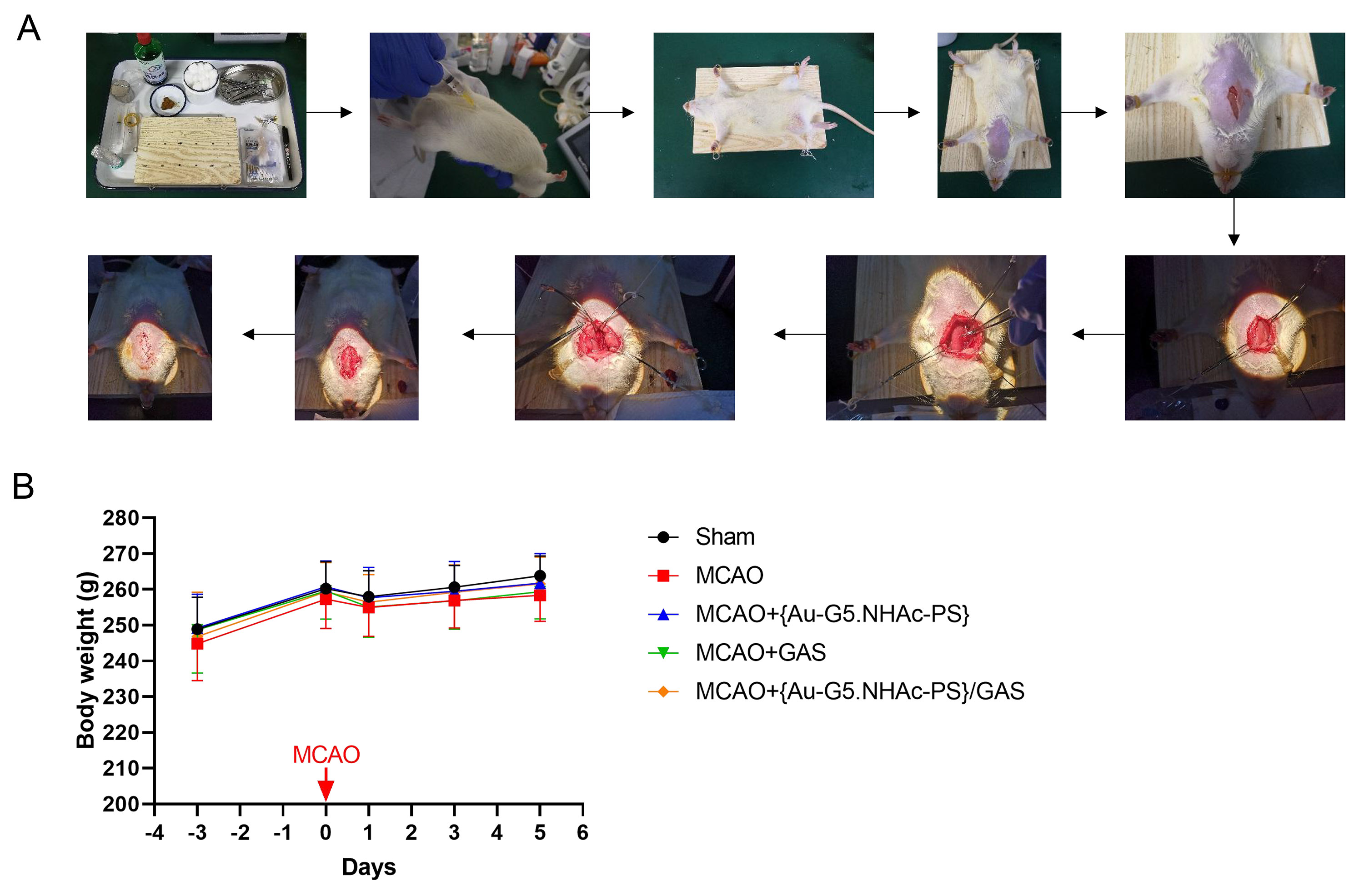

Supplement: Supplementary file 1 — FIGURE S1 (A) the flowchart of MCAO modeling in rats; (B) trends in body weight of rats in each group [file BRB3-12-e2810-s001.jpg]
